# Representation of Markush structures: from molecules toward patents

**DOI:** 10.1186/1758-2946-3-S1-O7

**Published:** 2011-04-19

**Authors:** Szabolcs Csepregi, Nóra Máté, Róbert Wágner, Tamás Csizmazia, Szilárd Dóránt, Erika Bíró, Tim Dudgeon, Ali Baharev, Ferenc Csizmadia

**Affiliations:** 1 ChemAxon Ltd., R&D, Máramaros köz 3/a, Budapest, 1037, Hungary

## 

Cheminformatics systems usually focus primarily on handling specific molecules and reactions. However, Markush structures are also indispensable in various areas, like combinatorial library design or chemical patent applications for the description of compound classes.

The presentation will discuss how an existing molecule drawing tool (Marvin) and chemical database engine (JChem Base/Cartridge) are extended to handle generic features (R-group definitions, atom and bond lists, link nodes and larger repeating units, position and homology variation). Markush structures can be drawn and visualized in the Marvin sketcher and viewer, registered in JChem databases and their library space is searchable without the enumeration of library members. Different enumeration methods allow the analysis of Markush structures and their enumerated libraries. These methods include full, partial and random enumerations as well as calculation of the library size. Furthermore, unique visualization techniques will be demonstrated on real-life examples that illustrate the relationship between Markush structures and the chemical structures contained in their libraries (involving substructures and enumerated structures).

Special attention will be given to file formats and how they were extended to hold generic features.

